# Adaptation of novel H7N9 influenza A virus to human receptors

**DOI:** 10.1038/srep03058

**Published:** 2013-10-28

**Authors:** J. C. F. M. Dortmans, J. Dekkers, I. N. Ambepitiya Wickramasinghe, M. H. Verheije, P. J. M. Rottier, F. J. M. van Kuppeveld, E. de Vries, C. A. M. de Haan

**Affiliations:** 1 Virology Division, Department of Infectious Diseases & Immunology, Faculty of Veterinary Medicine, Utrecht University, 3584 CL Utrecht, The Netherlands; 2 Pathology Division, Department of Pathobiology, Faculty of Veterinary Medicine, Utrecht University, 3584 CL Utrecht, The Netherlands

## Abstract

The emergence of the novel H7N9 influenza A virus (IAV) has caused global concerns about the ability of this virus to spread between humans. Analysis of the receptor-binding properties of this virus using a recombinant protein approach in combination with fetuin-binding, glycan array and human tissue-binding assays demonstrates increased binding of H7 to both α2-6 and α2-8 sialosides as well as reduced binding to α2-3-linked SIAs compared to a closely related avian H7N9 virus from 2008. These differences could be attributed to substitutions Q226L and G186V. Analysis of the enzymatic activity of the neuraminidase N9 protein indicated a reduced sialidase activity, consistent with the reduced binding of H7 to α2-3 sialosides. However, the novel H7N9 virus still preferred binding to α2-3- over α2-6-linked SIAs and was not able to efficiently bind to epithelial cells of human trachea in contrast to seasonal IAV, consistent with its limited human-to-human transmission.

Up until now (6/11/2013), H7N9 IAVs infected 133 and killed 39 people in China, although only few cases have been reported since the beginning of May 2013. The new human H7N9 virus is a reassortant virus that acquired its hemagglutinin (HA) subtype 7 (H7) and neuraminidase (NA) subtype 9 (N9) gene segments as well as the other gene segments from (different) avian IAVs^1^,^2^,^3^. Analysis of the gene sequences indicates that the novel H7N9 viruses may be better adapted to infect humans than other avian influenza A viruses. H7N9 viruses isolated from human possess for instance the E627K substitution in the PB2 RNA polymerase subunit, which has been shown to facilitate RNA replication in mammalian cells^4^,^5^,^6^ and transmission between ferrets^7^.

The specificity of the interaction of HA with sialic acids (SIAs), the cellular receptor, is a critical determinant of the host range of IAVs. In general, human viruses prefer binding to α2-6-linked sialosides, whereas avian viruses display a preference for SIAs linked to the penultimate galactose via α2-3-linkage^8^,^9^. The HA receptor binding site is formed by three structural elements that comprise the 130 loop (residues 133–138), the 190 helix (residues 190–198), and the 220 loop (residues 220–229)^10^. While avian viruses in general contain a glutamine at position 226 (H3 numbering), the human H7N9 virus has a leucine residue at this position. This amino acid substitution has been associated with reduced binding of avian receptors (α2,3-linked SIAs), increased binding of mammalian-like receptors (α2,6-linked SIAs)^8^,^11^,^12^, and with airborne transmission of H5N1 virus between ferrets^7^,^13^.

The NA protein plays an opposing role to HA as it removes SIA residues from glycans that are otherwise bound to HA^14^. The action of the NA protein thus promotes virus release after budding, but is also required for detachment of virus from decoy SIAs exposed e.g. on mucus. Although not much is known about the determinants of NA substrate specificity, it is generally accepted that the activity of NA needs to match the activity of HA to achieve efficient viral infection and replication^15^,^16^,^17^,^18^. It is at present not known to what extent the activity and specificity of the NA protein of the human H7N9 viruses contribute to infection of and replication in humans.

The human H7N9 virus was recently shown to replicate in the upper and lower respiratory tract of ferrets, the primary mammalian model for human influenza. The virus was furthermore efficiently transmitted via direct contact although less efficiently by airborne exposure^19^. In the present study, we compared the receptor-binding and enzymatic properties of the HA and NA proteins of the human H7N9 virus from 2013 with a closely related avian H7N9 virus. The results indicate that the novel H7N9 viruses have acquired the ability to bind to human-like receptors, for which substitutions at position 186 and 226 are responsible. These mutations are not only found in most novel human H7N9 viruses but are also found in the majority of novel avian H7N9 IAVs. Fortunately, however, the novel H7N9 viruses have not (yet) a receptor-binding profile resembling that of seasonal IAVs and are also not able to bind to the human tracheal epithelium. These results are in agreement with the limited spread of the novel H7N9 viruses between humans.

## Results

In the current study, we compared the receptor-binding properties of the H7 protein of the human H7N9 virus (referred to as human H7) with that of a closely related H7N9 virus derived from an Eurasian teal (*Anas crecca*; referred to as teal H7) using a recombinant protein approach^20^,^21^. A similar approach^22^ was used to compare the properties of the N9 proteins of these two viruses (referred to as human and teal N9). The recombinant protein approach renders the use of viruses, which results in obvious biosafety issues, superfluous. Recent studies have shown the potential of recombinant soluble oligomeric HA (sHA_3_) and NA (sNA_4_) proteins to study the receptor-binding properties of HA and the enzymatic activity of NA^17^,^20^,^21^. The genetic distance between the human and teal HA and NA proteins is shown in supplementary Fig. S1. The teal H7N9 virus is one of the closest relatives of the novel H7N9 viruses that contains both the H7 and the N9 genes. This was also exemplified by a protein blast search with the human H7 and N9 proteins, in which the teal H7 and N9 proteins were the first hits that are derived from a H7N9 virus sequenced prior to 2013. Comparison of functional pairs of HA and NA proteins of the teal and human H7N9 viruses not only allows us to study and compare the receptor-binding properties and enzymatic activities of H7 and N9 proteins derived from different hosts, but also to study the functional relationship between these complementary proteins.

### Human and teal H7 proteins differ in their fetuin binding

**Figure 1:**
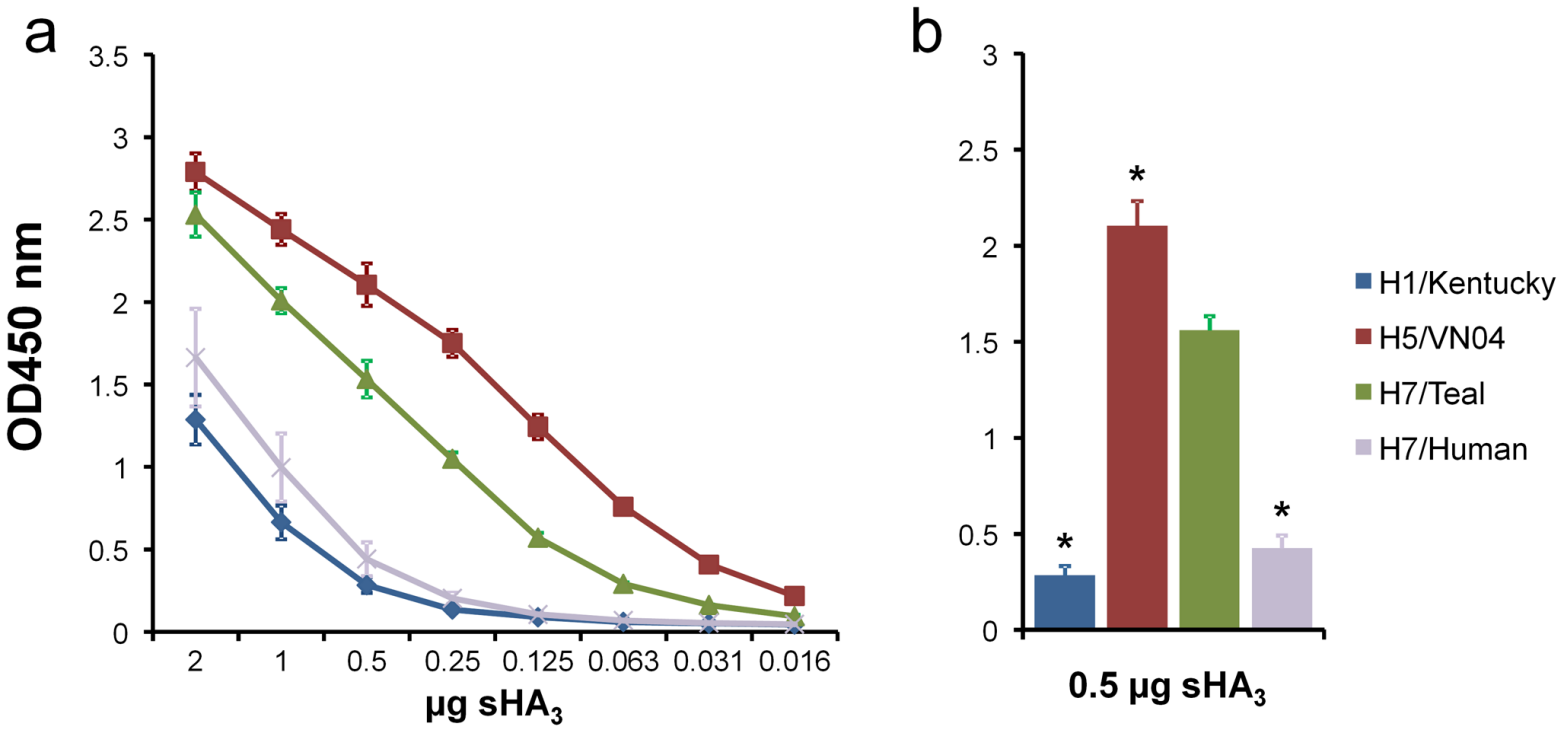
Fetuin binding of recombinant soluble trimeric HA proteins. (A) Limiting dilutions of soluble HA trimers (sHA_3_), complexed with horseradish peroxidase (HRP)-conjugated antibodies, were applied in the fetuin-binding assay. The optical density at 450 nm (OD 450) corresponds with binding of HA to fetuin. (B) Bar graph of the HA-fetuin binding at 0.5 μg HA protein. The mean values of at least two independent experiments performed in triplicate are shown Standard deviations are indicated, the asterisk (*) indicates a significant difference in binding between the human H7 (H7/Human) and the teal H7 (H7/Teal) protein (P < 0.001; One-way ANOVA followed by a Dunnett's multiple comparison test).

The purified human and teal H7 proteins were tested for their ability to bind fetuin. Fetuin contains mono-, bi-, and tri-antennary glycans carrying both α2-3 and α2-6 SIAs^23^. Binding of HA to fetuin was detected both for the teal and human H7 proteins, albeit with different efficiencies (Fig. 1A). The binding of the human H7 protein was significantly less efficient than that of the teal H7 protein (Fig. 1B). For comparison, the H5 (derived from an avian H5N1 virus) and H1 (derived from a seasonal human H1N1 virus) proteins, which were previously shown to specifically bind to α2-3 and α2-6 SIAs, respectively^21^,^24^, were also included. Binding of H5 to fetuin was more efficient than that of the H1 protein, which displayed similar binding to fetuin as the human H7 protein. We also analyzed the ability of the different recombinant proteins using hemagglutination assays with chicken, human or horse erythrocytes. (supplementary Fig. S2). The human H7 protein agglutinated both human and chicken erythrocytes, but to a lesser extent than the teal H7 or the H5 protein. The human H7 protein did not agglutinate horse erythrocytes. The H1 protein only showed appreciable agglutination of human erythrocytes. These results show that the human and teal H7 proteins have different receptor-binding properties. Whether this also reflects a difference in binding specificity (α2-3 versus alpha α2-6 SIA) will be addressed below. Furthermore, the hemagglutination assays indicate differences in receptor binding between the human H7 and H1 proteins.

### Human and teal N9 proteins differ in their enzymatic activity

**Figure 2:**
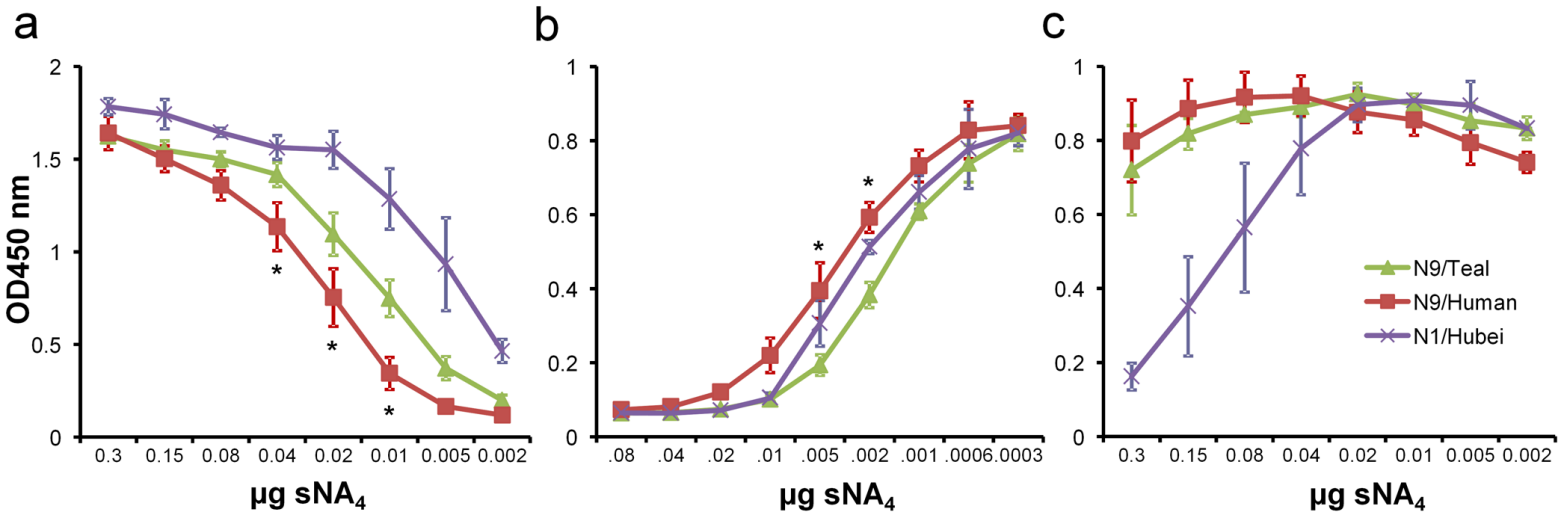
Activity and specificity of recombinant soluble tetrameric NA proteins. (A) Binding of biotinylated peanut agglutinin (PNA), which correlates with the amount of sialic acids released from galactose, after incubation of fetuin with limiting dilutions of soluble NA tetramers (sNA_4_). The specificity of the NA proteins was determined by measuring the binding of biotinylated MAL I, specific for α2-3 sialosides, (B) and SNA, specific for α2-6 sialosides (C) after incubation of fetuin with limiting dilutions of NA tetramers. The binding of PNA, MAL-I and SNA was detected using HRP-labeled streptavidin. The mean values of at least two independent experiments performed in triplicate are shown. Standard deviations are indicated, asterisks (*) indicate significant differences between the human N9 (N9/Human) and the teal N9 (N9/Teal) proteins (P < 0.001; One-way ANOVA followed by a Bonferroni test).

The purified NA proteins were analyzed for their enzymatic activity by assaying the ability of these proteins to remove SIA residues from fetuin, which results in the formation of peanut agglutinin (PNA) binding sites^25^ (Fig. 2A). The teal N9 protein consistently displayed a significantly higher sialidase activity than the human N9 protein and less N9 protein was needed for half maximal PNA binding (EC50 of 0.01380 vs 0.029 μg, P < 0.001). In agreement herewith, the teal protein more efficiently reduced the binding of the α2-3 SIA-specific lectin MAL I compared to the human protein (Fig. 2B). However, both proteins were equally inefficient in the removal of α2-6 SIAs, as incubation of fetuin with these proteins resulted in negligible reduction in binding of the α2-6 SIA-specific lectin SNA, compared to a control N1 protein (Fig. 2C). Thus, compared to the teal virus, the human H7N9 virus not only carries an HA protein that displays less efficient binding to fetuin, it also contains an NA protein that is less active.

### Residues at position 186 and 226 affect HA-receptor interactions

To identify the amino acids responsible for the observed differences in receptor binding between the human and teal H7 proteins, we performed site directed mutagenesis in the background of the teal protein. We focused on the region of the HA protein that comprises the 130 loop, 190 helix and 220 loop, which forms the receptor binding site. This region contains seven amino acid differences between the teal and human H7 proteins (supplementary Fig. S3). With the exception of the A/Shanghai/1/2013 (H7N9) virus, these seven substitutions were consistently found in all human H7N9 viruses. Also most novel avian H7N9 viruses contain these mutations.

**Figure 3:**
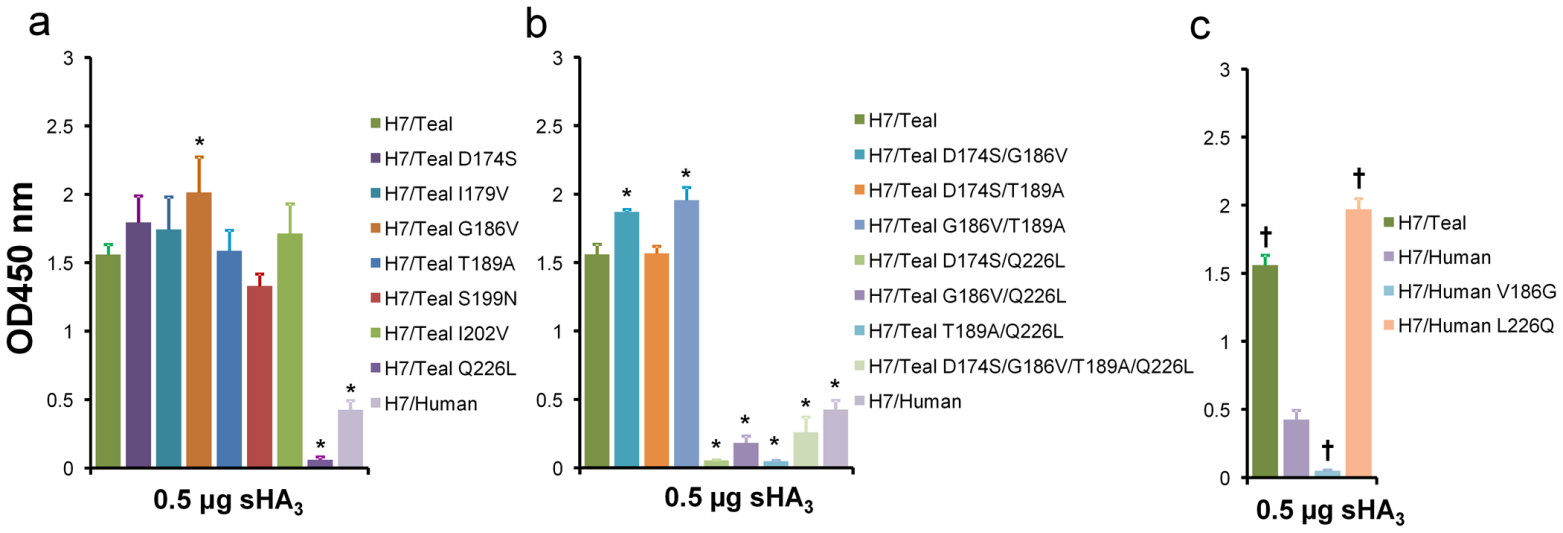
Fetuin binding of mutant recombinant soluble trimeric H7 proteins. Fetuin binding of recombinant soluble trimeric HA (sHA_3_) proteins carrying (A) single, (B) double or quadruple amino acid substitutions in H7/Teal or (C) single substitutions in H7/Human at 0.5 μg HA protein. The mean values of at least two independent experiments performed in triplicate are shown. Standard deviations are indicated and asterisks (*) and crosses (†) indicate significant differences in binding between (mutant) proteins and H7/Teal (A and B) or H7/Human (C), respectively (P < 0.001; One-way ANOVA followed by a Dunnett's multiple comparison test). Binding at different concentrations of HA is shown in supplementary Figure S4.

Teal H7 proteins that contained each of these mutations were expressed, purified, and tested for fetuin binding. Most mutations had little effect on the ability of the teal H7 protein to bind to fetuin. One mutation caused a modest but significant increase (G186V) in fetuin binding, while another mutation (Q226L) severely decreased the binding of the H7 protein (Fig. 3A and supplementary Fig. S4). Next, we tested different combinations of mutations. In general, amino acid substitutions in addition to those at position 186 or 226 did not affect the fetuin-binding properties of the resulting proteins much compared to the 186 and 226 single mutant proteins. Only when the mutations at position 186 and 226 were combined was a fetuin-binding phenotype similar to that of the human H7 protein observed. (Fig. 3B and supplementary Fig. S4). Binding of this double mutant protein was significantly higher than that of the Q226L single mutant protein (P < 0.001). Introduction of two additional substitutions in the H7 protein already carrying both G186V and Q226L did not significantly affect fetuin binding compared to the G186V and Q226L double mutant. In agreement with the important role for the residues at position 186 and 226 in the teal protein for fetuin binding, reciprocal substitutions at positions 186 and 226 in the background of the human H7 protein resulted in significantly decreased (V186G) and increased (L226Q) fetuin binding (Fig. 3C and supplementary Fig. S4). Thus, single substitutions at position 186 and 226 in the background of the human H7 protein are sufficient to obtain fetuin-binding properties that are similar to single mutant teal proteins (compare H7/Human L226Q with H7/Teal G186V [P > 0.7] and H7/Human V186G with H7/Teal Q226L [P > 0.3]). In general, the fetuin-binding properties of the recombinant proteins correlated with the ability of these proteins to agglutinate red blood cells (supplementary Fig. S2). Although we cannot exclude that other substitutions may also affect fetuin binding, we conclude that the differences in fetuin binding between the human and teal H7 proteins can be mostly attributed to substitutions at position 186 and 226.

### Glycan array analysis of H7 proteins

**Figure 4:**
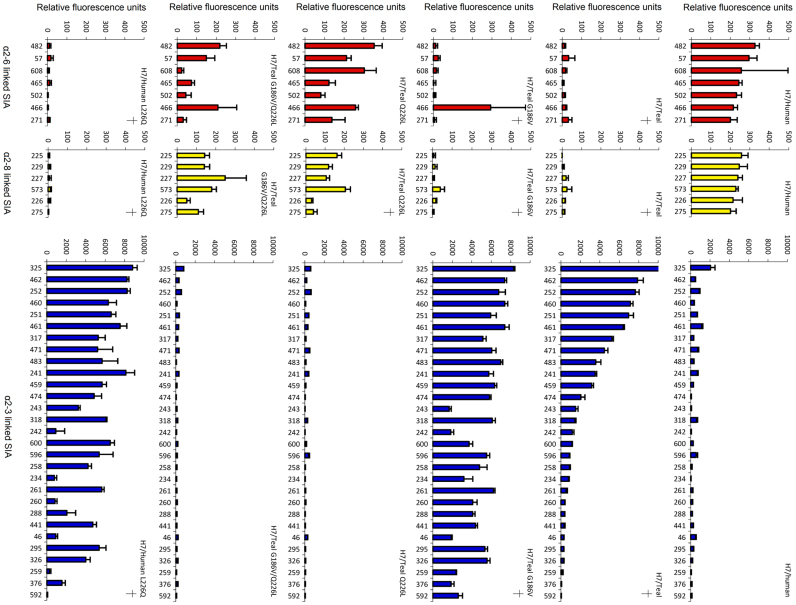
Glycan array analysis of H7 proteins. H7/Human, H7/Teal and the indicated single and double amino acid substitution mutants were applied to the glycan array. Soluble trimeric HA proteins (10 ug) were pre-complexed with anti-Strep tag mouse monoclonal antibody and fluorescent secondary antibodies as described^20^,^21^. Panels display binding to glycans carrying α2-6 linked (red), α2-8 linked (yellow) or α2-3 linked (blue) SIAs (full glycan array data will become available at www.functionalglycomics.org). Glycan numbers indicated on the X-axes correspond to the structures in supplementary table S1. Y-axes indicate relative fluorescence units. Glycans are sorted from left to right for highest binding to H7/human (α2-6 and α2-8 SIAs) or H7/teal (α2-3 SIAs) proteins. The mean values of an experiment performed in sixfold are shown. Crosses (†) indicate data sets (α2-6, α2-8, or α2-3 SIAs), the mean of which is significantly different from the mean of the data sets obtained with the H7/human protein (P < 0.001; One-way ANOVA followed by a Dunnett's multiple comparison test).

For a detailed analysis of the SIA-binding properties of the human and teal H7 proteins, the soluble trimeric HA preparations were subjected to glycan array analysis in collaboration with the Consortium for Functional Glycomics. In addition, several mutant H7 proteins were analyzed. As expected the teal H7 protein efficiently bound to a large number of α2-3 SIA-containing glycans (Fig. 4 and supplementary Table S1), but did not display any binding above background signals to human-like α2-6 SIA-containing receptors. The human H7 protein displayed significantly increased binding to a subset of α2-6 and surprisingly also α2-8-linked SIA-containing glycans when compared to the teal protein. In contrast, binding to α2-3 sialosides was strongly decreased but still stronger when compared to binding to α2-6-containing glycans. The H7 proteins did not display any appreciable binding to N-glycolyl sialosides.

Introduction of the Q226L mutation in the teal H7 protein resulted in decreased binding to α2-3 sialosides and increased binding to α2-6/α2-8 sialosides, resembling, apart from some quantitative differences, the patterns observed for the human H7 protein. Additional introduction of the G186V mutation did not drastically affect the shift in binding from α2-3 to α2-6/α2-8 sialosides although the extent to which several individual glycans were bound was affected by the double mutation (e.g. reduced binding of glycans 608 and 271). Introduction of only the G186V mutation in the teal H7 protein clearly increased binding of HA to a subset of α2-3 sialosides, in agreement with the fetuin assay. Introduction of the L226Q substitution in the human protein resulted in negligible binding to α2-6/α2-8 sialosides but resulted in increased binding to α2-3 SIA-containing glycans.

### Binding of HA to human tissues

**Figure 5:**
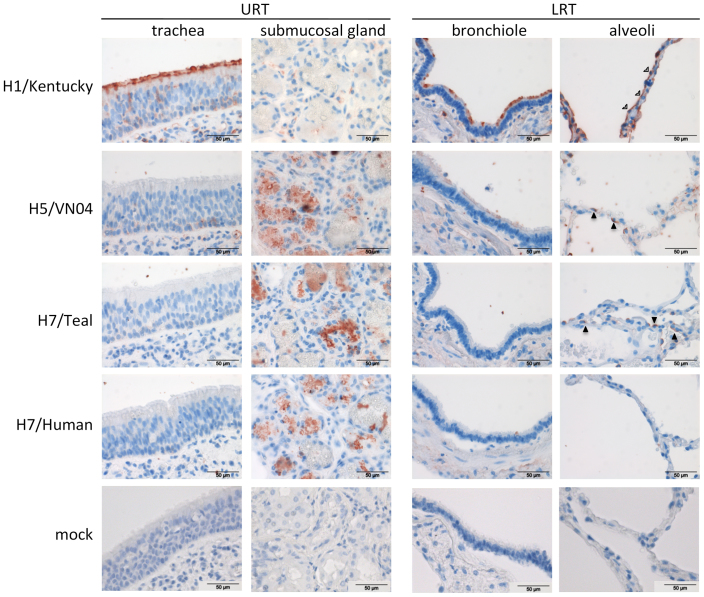
HA histochemistry. HA histochemistry was performed by incubating human trachea and lung tissues with the indicated HA proteins (H1/Kentucky: H1 protein derived from a human seasonal influenza H1N1 virus; H5/VN04; H5 protein from an avian H5N1 virus) after precomplexing with Streptactin-HRP as described previously^24^. Black arrowhead point to type II pneumocytes, while open arrowheads point to type I pneumocytes. URT: upper respiratory tract, LRT: lower respiratory tract. As a negative control, slides were incubated by Streptactin-HRP in the absence of HA (mock).

Next we studied the ability of the human and teal H7 proteins to bind to human tissues. Previously, others have shown that human viruses with a preference for α2-6 SIAs are able to bind to epithelial cells lining the upper respiratory tract (URT)^11^,^26^. These human viruses are also able to bind to cells of the lower respiratory tract (LRT). Viruses that prefer binding to α2-3 SIAs are able to bind to cells of the LRT, because of the presence of α2-3 SIAs in the LRT, but not to the epithelial cells of the URT. Indeed, comparable results were obtained by performing recombinant HA histochemistry on human tissues using the H1 and the H5 protein (Fig. 5). The H1/Kentucky protein of the seasonal influenza virus, which was previously shown by microarray analysis to bind to α2-6 SIAs only^21^, displayed efficient binding to the cilia of the human trachea epithelium. In the lung, the H1 protein bound to the ciliated epithelium of the bronchiole and to type I pneumocytes. In contrast, the H5 protein, which only binds to α2-3 SIAs^24^, did not demonstrate appreciable binding to the ciliated epithelium of the trachea, although efficient binding to submucosal glands was observed. In the LRT, the H5 protein displayed binding to the cilia of the bronchiole, to type II pneumocytes (Fig. 5) and occasionally to macrophages (data not shown). The teal H7 protein showed a similar binding pattern as the H5 protein and bound to submucosal glands and type II pneumocytes, consistent with the expression of α2-3 sialosides on these cells^27^. In contrast, the human H7 protein only showed efficient binding to the submucosal glands. Hardly any binding was observed to the cilia of the bronchiole and to pneumocytes, in agreement with the reduced receptor-binding avidity of this protein. No staining was observed in the absence of HA (mock).

## Discussion

Pandemics of influenza A virus are caused by viruses that suddenly appear from animal reservoirs, to which most humans have no immunity and which are able to efficiently transmit between humans. A prerequisite for efficient human-to-human transmission is the ability of the virus to specifically bind to α2-6-linked SIAs present in the upper respiratory tract. In view of the concerns raised by the novel human H7N9 virus we analyzed the receptor binding properties of this virus. The results indicate that, in comparison to avian H7N9 virus, the human H7N9 virus displays increased binding to α2-6 as well as α2-8 sialosides and reduced binding to α2-3-linked SIAs. Still, whereas all seasonal/pandemic IAVs bind more efficiently to α2-6- than to α2-3-linked sialosides, the human H7 protein binds more efficiently to α2-3- than α2-6-linked SIAs and is not able to efficiently bind to epithelial cells of human trachea. From these results we conclude that the human H7N9 virus has not (yet) adapted its HA protein to such an extent that it results in a receptor-binding profile similar to that of pandemic/seasonal IAV. Our results are in agreement with recent studies that use recombinant H7 proteins to study the ability of the H7N9 viruses to interact with sialosides or to bind to human tissues^28^,^29^,^30^. Also when virions are used for receptor-binding analyses, human H7 viruses display lower avidity for α2-3 sialic acids and a higher avidity for α2-6 sialosides when compared to other avian viruses. In general, binding to α2-6 sialosides appears less than what is observed for human seasonal influenza viruses, which are able to bind to α2-6 but not α2-3 sialosides^30^,^31^,^32^,^33^. These results are in agreement with the limited human-to-human transmission and airborne transmission between ferrets of the human H7N9 virus^31^,^33^,^34^.

The relevance of the human H7N9 virus to bind to α2-8 sialosides is at present unclear. Most IAVs do not appear to bind to these glycans, although binding to α2-8 sialosides has been reported for IAV A/NWS/33 (H1N1)^35^, IAV X31 (H3N2) that carries the HA gene of A/Aichi/2/68 from the 1968 pandemic^36^, and IAV A/California/04/2009 (H1N1)^36^. α2-8 sialosides are most abundant in neural cells^37^,^38^ but have more recently been identified on a number of proteins outside the nervous system, including the glycophorins of human erythrocytes^39^ and serum proteins like α2-macroglobulin, plasminogen and Ig light chain^40^. To our knowledge α2-8 sialosides have never been reported to be present on epithelial cells in the respiratory tract or to be able to serve as functional receptors leading to cell entry. Nevertheless, IAVs are likely to encounter α2-8 sialosides present on the molecules described above and thus their potential role as decoy receptor needs to be considered, especially since IAV neuraminidases are not able to cleave α2-8 linkages^35^.

Relative to the teal H7 protein, the human H7 displayed reduced binding to α2-3 and increased, but weak, binding to α2-6 sialosides. In agreement with the hypothesis that the activity of NA balances the activity of HA^15^,^16^,^17^,^18^, the human N9 protein was significantly less active than the corresponding teal protein, which could be attributed to a loss in the ability to target α2-3 SIA substrates. Both the teal and the human proteins were equally inefficient in the removal of α2-6 SIA residues. The relative inability of the human N9 protein to remove α2-6 SIA residues may negatively affect the ability of the H7N9 virus to obtain more efficient binding to these human-like receptors as efficient replication of such viruses probably requires adaptive mutations in the NA protein.

The residues at position 186 and 226 are the main residues responsible for the observed differences in receptor binding. Introduction of the Q226L mutation resulted in severely reduced fetuin binding and in decreased or increased binding of α2-3 and α2-6 sialosides, respectively. The reduced binding to fetuin was compensated by G186V. The observation that a (partial) switch in receptor specificity results in reduced binding of sialosides, which is compensated by mutations at other positions in the HA protein, also appears to occur for other HA proteins. Mutations in H5 required for airborne transmission between ferrets not only include mutations responsible for a switch in receptor specificity, but also the loss of a N-linked glycosylation site at position 158^7^,^13^. The loss of this glycosylation site results in enhanced binding of H5 to human-type receptors^41^ (de Vries and de Haan, unpublished data).

Whereas avian viruses are highly specific for binding to α2-3 sialosides, all human viruses that efficiently spread between humans or ferrets via respiratory droplet transmission display a complete switch to α2-6 receptor specificity. Strikingly, almost all novel avian H7N9 viruses already carry the Q226L and G186V mutations, indicating that mutations that increase binding to human-like receptors may be obtained by IAVs in an avian host. The increased ability of such viruses to replicate in humans may promote the acquirement of subsequent substitutions that increase α2-6 binding. Additional mutations, required for a full switch, have been described for H2, H3 and H5 viruses. While human H2 and H3 viruses have obtained the G228S substitution^42^, for H5 an alternative mutation (N224K) may also suffice^13^. A scenario of stepwise development of optimal binding has a precedent in the observed evolution of the H2N2 pandemic. H2N2 viruses isolated from humans during the first year (1957) of the “Asian” pandemic had not yet obtained the G228S substitution and show lower avidity towards human-like receptors^42^. Probably, these viruses had not yet acquired the optimal SIA-binding properties for efficient human-to-human transmission. Likewise, the inability of the novel H7N9 viruses to efficiently transmit between humans so far, is probably related with the incomplete switch in receptor specificity that we observed for this virus.

## Methods

### Genes, expression vectors, protein expression and purification

Codon optimized H7 or N9 ectodomain encoding cDNAs (Genscript, USA) of A/Anhui/1/2013 (GISAID Isolate EPI439507, referred to as H7/human and N9/human) and of A/Anas crecca/Spain/1460/2008 (GenBank accession no. CAY39406, referred to as H7/teal and N9/teal) were cloned into expression plasmids similarly as described previously^20^,^21^,^22^. The HA ectodomain (a.a. 19–518; H3 numbering) encoding sequences were cloned in frame with sequences encoding a C-terminal artificial GCN4 trimerization motif 43 and a Strep-tag for affinity purification (IBA GmbH). The NA head domain (a.a. 71–465) encoding sequence was preceded by sequences successively coding for an N-terminal signal sequence, a double Strep-tag and an artificial GCN4 tetramerization domain^43^. The expression plasmids containing the H5 gene of A/Viet Nam/1194/2004 (GenBank accession no. ACU65077.1) and the H1 gene of A/Kentucky/07 (GenBank accession no. CY028163) have been described earlier^21^,^44^. The N1 head domain of A/Hubei/1/2010 (GenBank CY098760) was cloned similarly as described above. Site directed mutagenesis of the HA or NA-encoding sequences was performed with the Quickchange II XL Site-Directed Mutagenesis Kit (Agilent Technologies). The HA and NA proteins were expressed in HEK293S GnT1(−) or HEK293T cells and purified from the cell culture supernatants as described previously^20^,^21^,^22^.

### HA binding assays

Receptor-binding of HA was analyzed using a fetuin solid-phase binding and hemagglutination assays^20^,^21^ and by performing HA histochemistry on formalin-fixed, paraffin-embedded human tissues similarly as described previously^24^. Glycan array analysis of the HA proteins was performed by the Core H of the Consortium for Functional Glycomics as described previously^20^,^21^. The fetuin-binding assays were performed at least twice in triplicate. The mean values of at least two independent experiments are shown. HA proteins were expressed in HEK293S GnT1(−) cells, except for the HA histochemistry, for which HA proteins expressed in HEK293T cells were used.

### NA activity assays

Activity of the NA proteins expressed in HEK293T cells was analyzed using a previously described fetuin-based assay^25^. In brief, fetuin-coated 96-well plates, were incubated with limiting dilutions of recombinant soluble NA protein for 2 hour at 37°C. After washing, the plates were incubated with biotinylated peanut agglutinin (PNA, 2.5 μg/ml; Galab Technologies). Binding of PNA correlates with the amount of SIA released from galactose. The specificity of the NA proteins was determined by measuring the binding of biotinylated SNA or MAL I (both from Galab Technologies) after incubation of fetuin with NA. The binding of PNA, SNA and MAL-I was detected using horseradish peroxidase-labeled streptavidin. NA activity assays were performed at least twice in triplicate. The mean values of at least two independent experiments are shown. The amount of NA protein required for half maximum PNA binding (EC50) was determined using the GraphPad Prism software.

## Author Contributions

J.C.F.M.D., J.D., A.W., M.H.V. and E.d.V. performed the experiments. J.C.F.M.D., M.H.V., E.d.V., P.J.M.R., F.J.M.v.K., E.d.V. and C.A.M.d.H. designed the experiments. C.A.M.d.H. wrote the manuscript. All authors reviewed the manuscript.

## Supplementary Material

Supplementary Information supplementary data
