# Supplementary material for: Adaptation of novel H7N9 influenza A virus to human receptors
Source: Sci Rep. 2013 Oct 28;3:3058. doi: 10.1038/srep03058 (PMC3808826; doi:10.1038/srep03058)
Supplement: Supplementary Information — supplementary data [file srep0305-s1.pdf]

# ADAPTATION OF NOVEL H7N9 INFLUENZA A VIRUS TO HUMAN RECEPTORS

**Supplementary files.**

J.C.F.M. Dortmans[1], J. Dekkers[1], I. N. Ambepitiya Wickramasinghe[2], M.H. Verheije[2], P.J.M. Rottier[1], F.J.M. van Kuppeveld[1], E. de Vries[1], and C.A.M. de Haan[1]*

[1] Virology Division, Department of Infectious Diseases & Immunology, Faculty of Veterinary Medicine, Utrecht University, 3584 CL Utrecht, The Netherlands

[2] Pathology Division, Department of Pathobiology, Faculty of Veterinary Medicine, Utrecht University, 3584 CL Utrecht, The Netherlands

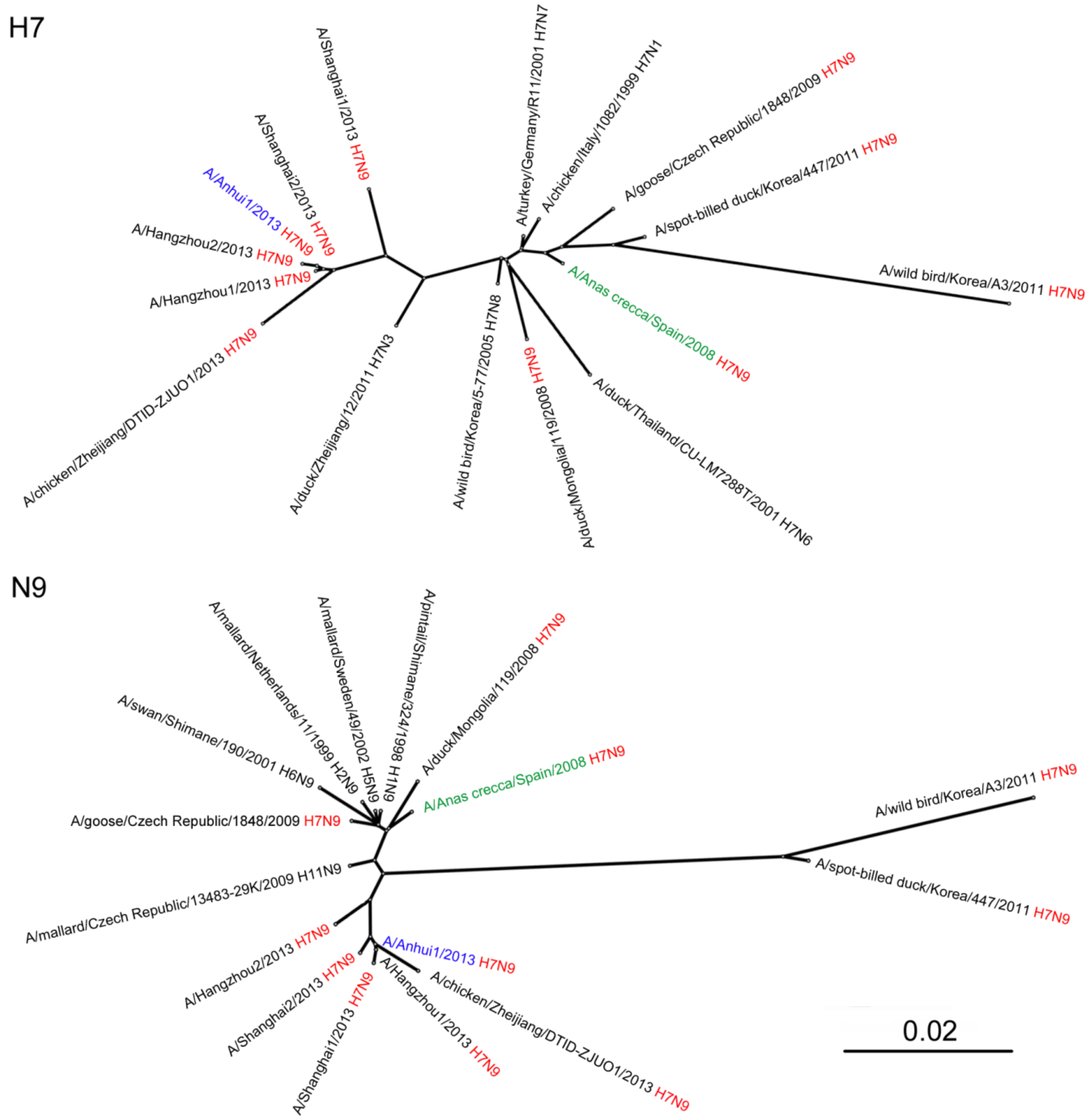


**Figure S1. Unrooted protein trees.**
Unrooted protein trees were constructed from full length H7 or N9 sequences using the Phylip Neighbor Joining algorithm from within the Ugene package (Dayhoff PAM distance matrix). Host species and year of isolation can be derived from the sequence names. H7N9 serotypes are labeled in red, other serotypes (H7Nx, HxN9) are in black. The proteins analyzed in this paper are derived from A/Anhui/1/2013 (blue) and A/Anas crecca/Spain/2008 (green).

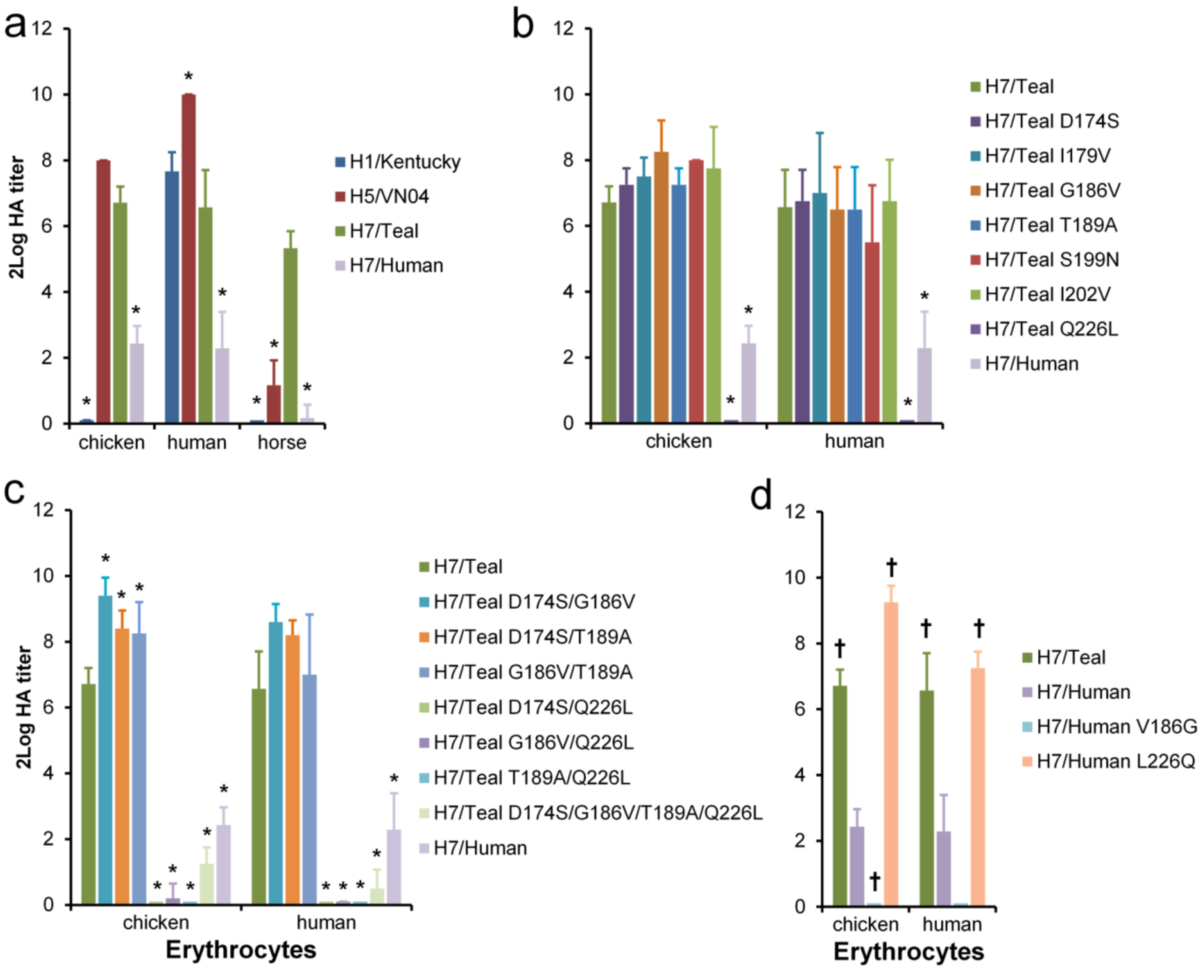


**Figure S2. Hemagglutination assay.**
Hemagglutination titers of the different pre-complexed soluble trimeric H7 proteins and mutants thereof were determined using either chicken, human or horse erythrocytes using 1 ug of HA as starting point as described previously[1]. Human and chicken erythrocytes contain both α2-6 and α2-3 sialic acids, although in different relative quantities (human cells contain more α2-6 sialosides than chicken cells[2]. Horse erythrocytes contain hardly any α2-6 sialic acids, while the majority of their α2-3 sialosides are of the N-glycolyl type (NeuGc), which is not present on human or chicken cells, which only contain sialosides of the N-Acetyl type (NeuAc). Nevertheless, viruses that prefer binding to α2-3 NeuAc are also able to agglutinate horse erythrocytes[3]. The mean values of at least two independent experiments performed in triplicate are shown. Standard deviations are indicated and asterisks (*) and crosses (†) indicate significant differences in HA titer between (mutant) proteins and H7/Teal (A, B and C) or H7/Human (D), respectively ($P<0.001$; One-way ANOVA followed by a Dunnett's multiple comparison test).

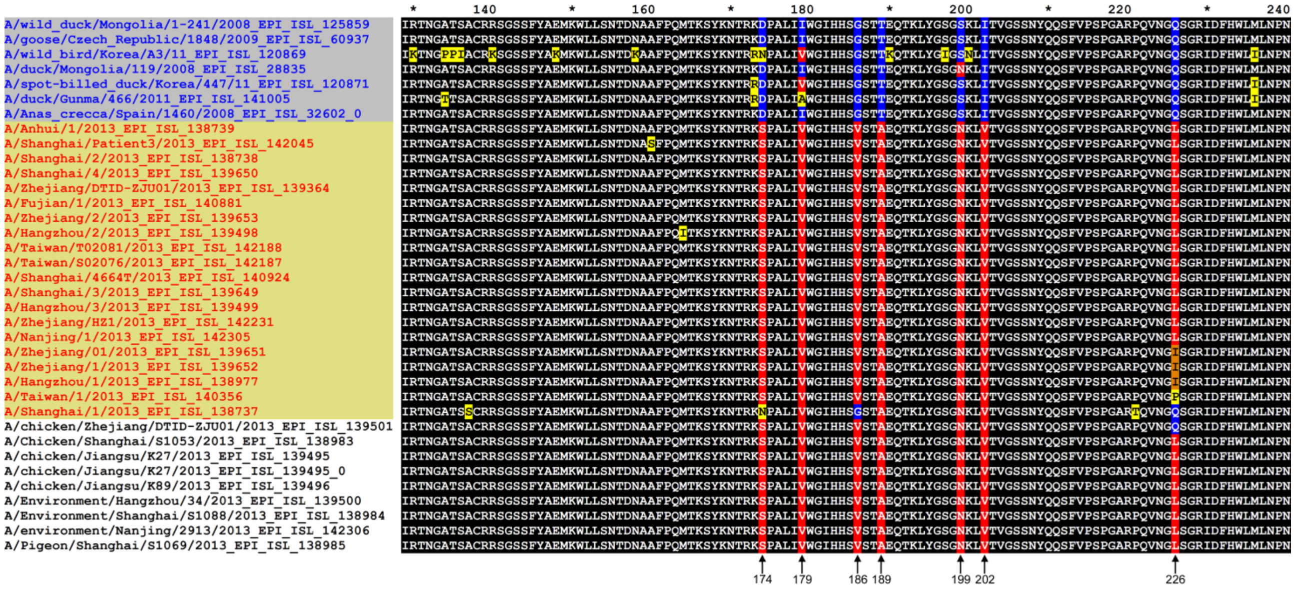


**Figure S3. Alignment of H7 proteins.**

Alignment of the H7 receptor binding site (residues 129-240, H3 numbering) of human (yellow shaded) and avian H7N9 virus isolates from 2013 and avian H7N9 virus isolates before 2013 (grey shaded). The seven positions that characterize the 2013 viruses are indicated by shading in red (H7N9 2013 consensus) or blue (H7N9 avian consensus before 2013). Other rare substitutions are shaded in yellow.

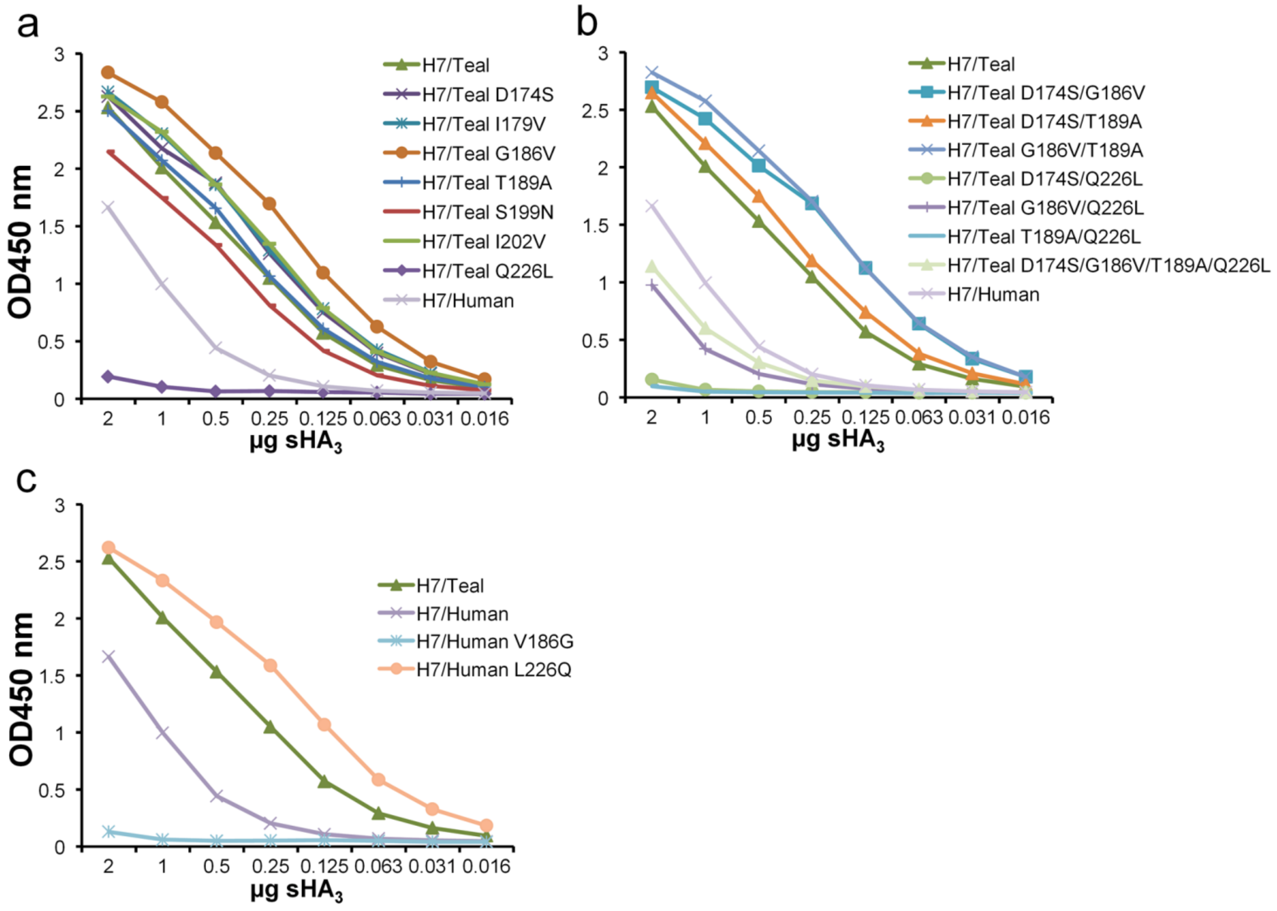


**Figure. S4. Fetuin-binding assay.**
Limiting dilutions of pre-complexed soluble trimeric H7 proteins and mutants thereof were applied in the fetuin-binding assay as described in the legend of Figure 1. The mean values of at least two independent experiments performed in triplicate are shown. The results shown correspond with the data shown in Figure 3.

## Table S1. List of glycans shown in Fig. 4.

| Glycan nr | Linkage type | Glycan structure |
|---|---|---|
| 57 | 2-6 | Neu5Aca2-6Galb1-4GlcNAcb1-2Mana1-6(Neu5Aca2-6Galb1-4GlcNAcb1-2Mana1-3)Manb1-4GlcNAcb1-4GlcNAcb-Sp24 |
| 271 | 2-6 | Neu5Aca2-6Galb1-4GlcNAcb1-3Galb1-4GlcNAcb-Sp0 |
| 465 | 2-6 | Neu5Aca2-6Galb1-4GlcNAcb1-6(Neu5Aca2-6Galb1-4GlcNAcb1-2)Mana1-6(GlcNAcb1-4)(Neu5Aca2-6Galb1-4GlcNAcb1-2Mana1-3)Manb1-4GlcNAcb1-4GlcNAcb-Sp21 |
| 466 | 2-6 | Neu5Aca2-6Galb1-4GlcNAcb1-6(Neu5Aca2-6Galb1-4GlcNAcb1-2)Mana1-6(GlcNAcb1-4)(Neu5Aca2-6Galb1-4GlcNAcb1-4(Neu5Aca2-6Galb1-4GlcNAcb1-2)Mana1-3)Manb1-4GlcNAcb1-4GlcNAcb-Sp21 |
| 482 | 2-6 | Neu5Aca2-6Galb1-4GlcNAcb1-2Mana1-6(Neu5Aca2-6Galb1-4GlcNAcb1-2Mana1-3)Manb1-4GlcNAcb1-4(Fuca1-6)GlcNAcb-Sp24 |
| 502 | 2-6 | Neu5Aca2-6GalNAcb1-4(6S)GlcNAcb-Sp8 |
| 608 | 2-6 | Neu5Aca2-6Galb1-4GlcNAcb1-3Galb1-4GlcNAcb1-2Mana1-6(Neu5Aca2-6Galb1-4GlcNAcb1-3Galb1-4GlcNAcb1-2Mana1-3)Manb1-4GlcNAcb1-4GlcNAcb-Sp12 |
| 225 | 2-8 | GalNAcb1-4(Neu5Aca2-8Neu5Aca2-8Neu5Aca2-8Neu5Aca2-3)Galb1-4Glcb-Sp0 |
| 226 | 2-8 | GalNAcb1-4(Neu5Aca2-8Neu5Aca2-8Neu5Aca2-3)Galb1-4Glcb-Sp0 |
| 227 | 2-8 | Neu5Aca2-8Neu5Aca2-8Neu5Aca2-3Galb1-4Glcb-Sp0 |
| 229 | 2-8 | Neu5Aca2-8Neu5Aca2-8Neu5Aca-Sp8 |
| 275 | 2-8 | Neu5Aca2-8Neu5Aca-Sp8 |
| 573 | 2-8 | Neu5Aca2-8Neu5Aca2-3Galb1-3GalNAcb1-4(Neu5Aca2-3)Galb1-4Glc-Sp21 |
| 46 | 2-3 | Neu5Aca2-3(6S)Galb1-4GlcNAcb-Sp8 |
| 234 | 2-3 | Neu5Aca2-3Galb1-3GalNAcb1-4(Neu5Aca2-3)Galb1-4Glcb-Sp0 |
| 241 | 2-3 | Neu5Aca2-3Galb1-4(Neu5Aca2-3Galb1-3)GlcNAcb-Sp8 |
| 242 | 2-3 | Neu5Aca2-3Galb1-3(6S)GalNAca-Sp8 |
| 243 | 2-3 | Neu5Aca2-6(Neu5Aca2-3Galb1-3)GalNAca-Sp8 |
| 251 | 2-3 | Neu5Aca2-3Galb1-4(6S)GlcNAcb-Sp8 |
| 252 | 2-3 | Neu5Aca2-3Galb1-4(Fuca1-3)(6S)GlcNAcb-Sp8 |
| 258 | 2-3 | Neu5Aca2-3Galb1-4GlcNAcb1-3Galb1-4GlcNAcb1-3Galb1-4GlcNAcb-Sp0 |
| 259 | 2-3 | Neu5Aca2-3Galb1-4GlcNAcb-Sp0 |
| 260 | 2-3 | Neu5Aca2-3Galb1-4GlcNAcb-Sp8 |
| 261 | 2-3 | Neu5Aca2-3Galb1-4GlcNAcb1-3Galb1-4GlcNAcb-Sp0 |
| 288 | 2-3 | Neu5Aca2-3Galb1-4GlcNAcb1-6(Galb1-3)GalNAca-Sp14 |
| 295 | 2-3 | Neu5Aca2-3Galb1-4GlcNAcb1-3Galb1-3GlcNAcb-Sp0 |
| 317 | 2-3 | Neu5Aca2-3Galb1-4GlcNAcb1-6(Neu5Aca2-3Galb1-3)GalNAca-Sp14 |
| 318 | 2-3 | Neu5Aca2-6Galb1-4GlcNAcb1-2Mana1-6(Neu5Aca2-3Galb1-4GlcNAcb1-2Mana1-3)Manb1-4GlcNAcb1-4GlcNAcb-Sp12 |
| 325 | 2-3 | Neu5Aca2-3Galb1-4GlcNAcb1-2Mana1-6(Neu5Aca2-3Galb1-4GlcNAcb1-2Mana1-3)Manb1-4GlcNAcb1-4GlcNAcb-Sp12 |
| 326 | 2-3 | Neu5Aca2-3Galb1-4GlcNAcb1-2Mana1-6(Neu5Aca2-6Galb1-4GlcNAcb1-2Mana1-3)Manb1-4GlcNAcb1-4GlcNAcb-Sp12 |
| 376 | 2-3 | Neu5Aca2-3Galb1-4GlcNAcb1-3GalNAc-Sp14 |
| 441 | 2-3 | Neu5Aca2-3Galb1-4GlcNAcb1-3Galb-Sp8 |
| 459 | 2-3 | Neu5Aca2-3Galb1-4GlcNAcb1-2Mana1-6(GlcNAcb1-4)(Neu5Aca2-3Galb1-4GlcNAcb1-2Mana1-3)Manb1-4GlcNAcb1-4GlcNAcb-Sp21 |
| 460 | 2-3 | Neu5Aca2-3Galb1-4GlcNAcb1-4Mana1-6(GlcNAcb1-4)(Neu5Aca2-3Galb1-4GlcNAcb1-4(Neu5Aca2-3Galb1-4GlcNAcb1-2)Mana1-3)Manb1-4GlcNAcb1-4GlcNAcb-Sp21 |
| 461 | 2-3 | Neu5Aca2-3Galb1-4GlcNAcb1-6(Neu5Aca2-3Galb1-4GlcNAcb1-2)Mana1-6(GlcNAcb1-4)(Neu5Aca2-3Galb1-4GlcNAcb1-2Mana1-3)Manb1-4GlcNAcb1-4GlcNAcb-Sp21 |
| 462 | 2-3 | Neu5Aca2-3Galb1-4GlcNAcb1-6(Neu5Aca2-3Galb1-4GlcNAcb1-2)Mana1-6(GlcNAcb1-4)(Neu5Aca2-3Galb1-4GlcNAcb1-4(Neu5Aca2-3Galb1-4GlcNAcb1-2)Mana1-3)Manb1-4GlcNAcb1-4GlcNAcb-Sp21 |
| 471 | 2-3 | Neu5Aca2-3Galb1-4GlcNAcb1-6(Neu5Aca2-3Galb1-4GlcNAcb1-3)GalNAca-Sp14 |
| 474 | 2-3 | Neu5Aca2-3Galb1-3GlcNAcb1-6(Neu5Aca2-3Galb1-3GlcNAcb1-2)Mana1-6(Neu5Aca2-3Galb1-3GlcNAcb1-2Mana1-3)Manb1-4GlcNAcb1-4GlcNAcb-Sp19 |
| 483 | 2-3 | Neu5Aca2-3Galb1-4GlcNAcb1-2Mana1-6(Neu5Aca2-3Galb1-4GlcNAcb1-2Mana1-3)Manb1-4GlcNAcb1-4(Fuca1-6)GlcNAcb-Sp24 |
| 592 | 2-3 | Neu5Aca2-3Galb1-4GlcNAcb1-3Galb1-4GlcNAcb1-3GalNAca-Sp14 |
| 596 | 2-3 | Neu5Aca2-3Galb1-4GlcNAcb1-3Galb1-4GlcNAcb1-6(Neu5Aca2-3Galb1-4GlcNAcb1-3Galb1-4GlcNAcb1-3)GalNAca-Sp14 |
| 600 | 2-3 | Neu5Aca2-3Galb1-4GlcNAcb1-3Galb1-4GlcNAcb1-6(Galb1-3)GalNAca-Sp14 |

## References


1. de Vries, R.P. et al. Only two residues are responsible for the dramatic difference in receptor binding between swine and new pandemic H1 hemagglutinin. *J. Biol. Chem.* **286**, 5868-5675 (2011).
2. Medeiros, R., Escriou, N., Naffakh, N., Manuguerra, J.C. & van der Werf, S. Hemagglutinin residues of recent human A(H3N2) influenza viruses that contribute to the inability to agglutinate chicken erythrocytes. *Virology* **289**, 74-85 (2001).
3. Ito, T. et al. Receptor specificity of influenza A viruses correlates with the agglutination of erythrocytes from different animal species. *Virology* **227**, 493-499 (1997).
